# An annotated checklist of the Crambidae of the region of Murcia (Spain) with new records, distribution and biological data (Lepidoptera: Pyraloidea, Crambidae)

**DOI:** 10.3897/BDJ.9.e69388

**Published:** 2021-08-03

**Authors:** Manuel J. Garre, John Girdley, Juan J. Guerrero, Rosa M. Rubio, Antonio S. Ortiz

**Affiliations:** 1 Universidad de Murcia, Murcia, Spain Universidad de Murcia Murcia Spain

**Keywords:** Lepidoptera, Crambidae, checklist, chorology, distribution, new records, phenology, Iberian Peninsula

## Abstract

**Background:**

The Murcia Region (osouth-eastern Iberian Peninsula) has a great diversity of Lepidopteran fauna, as a zoogeographical crossroads and biodiversity hotspot with more than 850 butterflies and moth species recorded.

**New information:**

In the present paper, based on an examination of museum specimens, published records and new samples, a comprehensive and critical species list of Crambidae moths (Lepidoptera: Pyraloidea) is synthesised. In total, 8 subfamilies, 50 genera and 106 species have been recorded and these are listed along with their collection, literature references and biological data including chorotype, voltinism and the flight period in the study area. The subfamilies are as follows: Acentropinae, Crambinae, Glaphyriinae, Lathrotelinae, Odontiinae, Pyraustinae, Scopariinae and Spilomelinae. Forty nine species are here newly recorded for the Murcia Region.

## Introduction

The Crambidae, belonging to the superfamily Pyraloidea, are mainly nocturnal micromoths (Microlepidoptera) with an estimated 10,100 named species worldwide, of which the European fauna is represented by ca. 490 species (Leraut 2012). In the Iberian Peninsula, 256 species have been recorded (Vives-Moreno 2014). The two main evolutionary lineages within Pyraloidea, Pyralidae and Crambidae, are monophyletically distinguished by the morphology of tympanal organs (Slamka 2008, Minet 1982, Minet 1983). Crambidae are characterised by the forewing venation with R5 free and an oval sclerotisation costad on base of vein A_1+2_; presence of a paired tympanal organs situated ventrally in the second abdominal segment with tympanum and conjunctivum at an obtuse angle, tympanal chamber cephalad open and accessory tympana in metathorax; lobulus and praecinctorium are present; male genitalia without uncus arms; and segment A8 of larvae without sclerotised ring around base of seta SD1 (Goater et al. 2005, Slamka 2008).

The Crambidae of Europe have been relatively well studied although there is a need for further investigation on habitus and distribution. The Southern European and especially Iberian species are poorly recorded and more precise data are necessary for the production of distribution maps.

Historically, the first crambid moth recorded and described from Murcia Region was *Ananiamurcialis* (Ragonot, 1895). Caradja (1910) recorded *Euchromiusramburiellus* (Duponchel) and *Ancylolomiadisparalis* (Hübner) and Zerny (1914) recorded *Udeainstitalis* (Hübner), *U.bipunctalis* (Herrich-Schäffer), *Mecynatrinalis* (Denis & Schiffermüller), *Metasiahymenalis* Guenée, *M.cuencalis* Ragonot, *Chrysoteuchiaculmella* (Linnaeus), *Loxostegecomptalis* (Freyer), *L.clathralis* (Hübner) and *Ephelispudicalis* (Duponchel). Subsequently, Caradja (1916) confirmed some of these species and recorded *Pediasiaribbeellus* (Caradja) and *Evergestisdesertalis* (Hübner). Bleszyński (1957) firstly recorded *Mesocrambuspallidellus* (Duponchel) and then Bleszyński (1959) also reported *Agriphilatrabeatellus* (Herrich-Schäffer).

Later, Agenjo (1952) confirmed and added new records of *Udeaferrugalis* (Hübner), *Dolicharthriabruguieralis* (Duponchel), *Ecpyrrhorrhoediffusalis* (Guenée), *Pyraustasanguinalis* (Linnaeus), *Ostrinianubilalis* (Hübner), *Titaniotarraconensis* Leraut & Luquet (cited as *Titanionormalis* (Hübner, 1796)), *Aporodesfloralis* (Hübner), *Hyperlaislutosalis* (Mann) and *Loxostegesticticalis* (Linnaeus), while Agenjo (1963) recorded *Ancylolomiatentaculella* (Hübner) and *Pseudoctenellainornata* Staudinger and Agenjo (1974) recorded *Pyraustapurpuralis* (Linnaeus).

Subsequent contributions are those of Derra and Hacker (1982), De Prins (1985), Schouten (1992), Pérez de-Gregorio (2006), Pérez de-Gregorio and Requena (2008), Pérez de-Gregorio and Requena (2010), Palacios and Abad (2010), Slamka (2010), Slamka (2013), Pérez de-Gregorio et al. (2011), Gastón et al. (2015), Leraut (2012) and Fazekas (2017).

The Region of Murcia has a great diversity of Lepidopteran fauna, as a zoogeographical crossroads and biodiversity hotspot, with more than 850 butterfly and moth species (Ortiz et al. 2016, unpublished data). The study area is located in the southeast of the Iberian Peninsula with an area of 11,313 km^2^ extending along the eastern end of the Betic Cordilleras, with ca. 27% of its surface corresponding to mountainous reliefs (Los Obispos peak, at 2,017 m a.s.l. as the highest elevation), 38% interior depressions and valleys and the remaining 35% plateaus and coastal plains. This variety of landscapes contributes to a great diversity of Lepidopteran fauna. The Region comprises areas of xeric-mediterranean and desertic climate, with high temperatures and low rainfall, making this territory one of the warmest and driest in Europe.

Temperature and precipitation are climatic factors which have a direct impact on the diversity and distribution of plants and the physiognomy of the plant landscape and are fundamental to the interpretation of the lepidopteran fauna of the Murcia Region.

**Figure 1.**
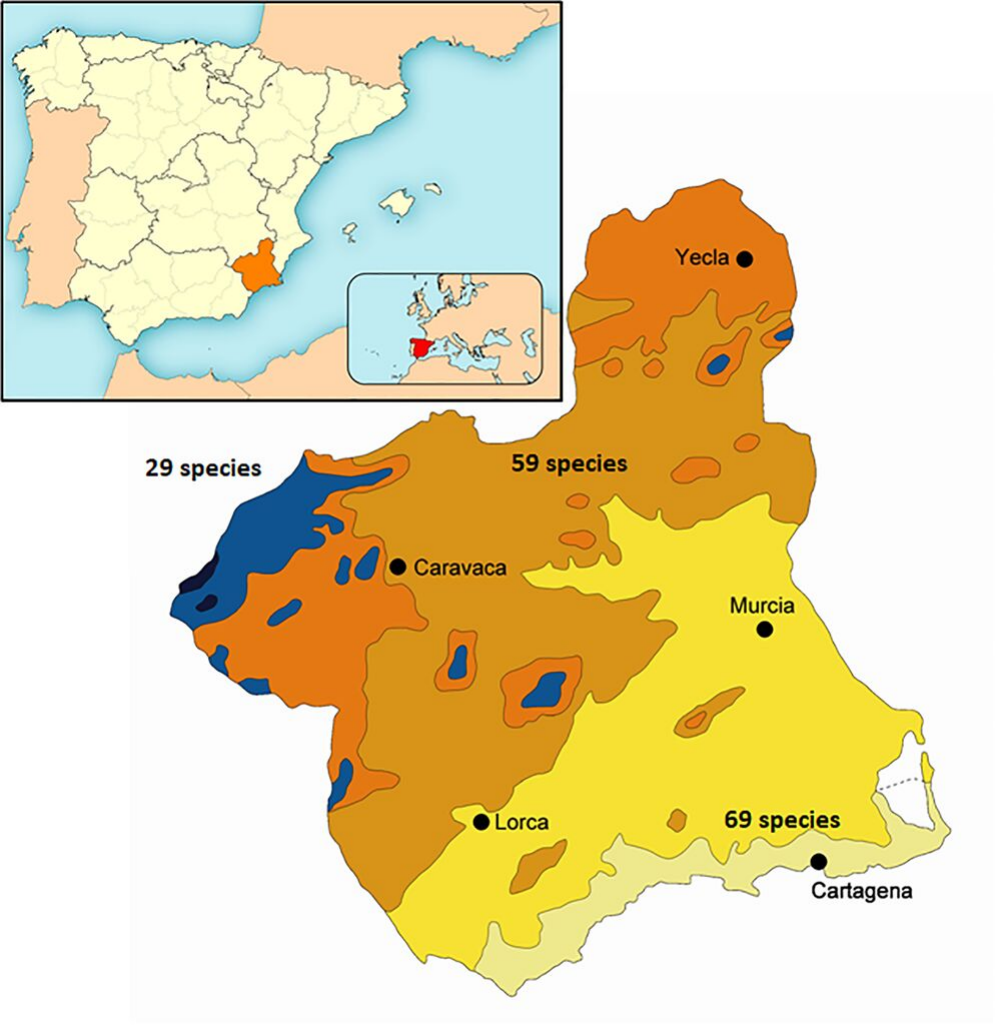
Map of the known species diversity in the bioclimatic areas in the Murcia Region. Black and blue: Oro- and Supramediteranean; orange and light brown: Cold and mild Mesomediterranean; Yelow and light green: Upper and lower Thermomediterranean.

Considering various bioclimatic approaches relative to temperature (thermotypes) and rainfall (ombrotypes), four different bioclimatic areas can be recognised according to Alcaraz et al. (2008): thermo-, meso-, supra- and oromediterranean (Fig. 1). Climatic and geological interactions differentiate a great variety of habitats as thermoxerophylic on the sunny slopes of the mountains and, on the other hand, as mesophylic in depressions or very dark exposures, in riparian zones amongst halophytic vegetation and on sandbanks and dunes from the inland to the coastal areas along with agricultural crops and anthropophilic areas. Altogether, they make up ten habitats and 47 special terrestrial conservation areas of community importance (Alcaraz et al. 2008).

This present checklist is intended to update the recorded species and to facilitate access to the most recent data on the Crambidae family from the Murcia Region (south-eastern Iberian Peninsula) for taxonomists providing data about distribution, chorology, phenology and voltinism.

## Materials and methods

The list contains all species of Crambidae collected by the authors until the end of 2020, along with the material deposited in the private collections of J.A. de la Calle, F. Lencina, F. Albert and F. Arcas. It also includes all of those records previously referenced in the bibliography.

Black and actinic (6 and 15 W) Heath traps, 125 W Robinson traps, 125 W mercury vapour traps and 4 W LED light traps were used for nocturnal sampling. Catches taken during daytime and in the urban environment (street lighting) are also included. All these sampling points are located within the study area and, especially, in the natural protected areas like the mountainous Parks of Sierra Espuña, Sierra de la Pila, El Valle and Carrascoy, etc. and the coastal Parks of Calblanque, Monte de las Cenizas and Peña del Águila, Salinas and Arenales de San Pedro del Pinatar, etc.

### Notes on the checklist

The subfamilies are systematically ordered and identified, based on the most recent classification of Crambidae by Léger et al. (2020), Slamka (2008) and Vives-Moreno (2014) with minor modifications. The genera and species are listed under their subfamilies and are also ordered systematically, together with collection data (sampling localities, altitude, decimal coordinates, date, number of specimens). In addition, for each species, related references and biological data are provided including chorotype, voltinism and the flight period in the study area indicated by months in Roman numerals. All studied specimens are deposited in the entomological collection in the Zoology Department of Murcia University (Spain) and in the collections of Francisco Lencina, Fernando Albert and Francisco Arcas. The occurrence data can be accessed at DOI: https://doi.org/10.15470/kffxc0

Goater et al. (2005), Slamka (2006), Slamka (2008), Slamka (2013) and Leraut (2012) were consulted to obtain the information on biology, voltinism and geographical distribution of the species, while Calle (1982) and Varga (2010) were consulted for biogeographic criteria.

## Checklists

### Annotated checklist of Crambidae recorded in the Murcia Region

#### Crambidae

BF352E75-7113-547A-A0DD-AE2883DC2C6C

#### Acentropinae

EC65CB17-DD7D-5BCF-8DAA-8CC903DB7CF1

#### Elophila nymphaeata

(Linnaeus, 1758)

D629F7D8-050A-538E-873E-969C5A093C28

##### Distribution

Eurasiatic

##### Notes

References: Speidel (1983). Biological data: Bivoltine.

#### Crambinae

31367547-7C41-59EC-AD35-5EFBFF53EF9A

#### Chilo phragmitellus

(Hübner, [1810])

E3122648-2902-5603-8D87-57017C86FAD8

##### Distribution

Eurasiatic

##### Notes

References: Palacios and Abad (2010). Biological data: Bivoltine.

#### Chilo luteellus

(Motschulsky, 1866)

9305ADBA-2551-5191-89D8-E80CDF712F0A

##### Distribution

Eurasiatic

##### Notes

Biological data: Univoltine. Flight period: V-VIII. First record in Murcia Region.

#### Pseudobissetia terrestrellus

(Christoph, 1885)

58F8E2FC-9A88-5222-8B1F-B94FAB792669

##### Distribution

Mediterranean-Asiatic

##### Notes

Biological data: Bivoltine. Flight period: VI. First record in Murcia Region.

#### Euchromius ocellea

(Haworth, 1811)

5A1231EF-EB89-5B40-8E83-109252F06901

##### Distribution

Cosmopolitan

##### Notes

References: De Prins (1985). Biological data: Bivoltine. Flight period: V-IX.

#### Euchromius rayatellus

Amsel, 1949

6EE6E8CD-6DF7-5E9F-91B1-E5227AFD9C7D

##### Distribution

Mediterranean-Asiatic

##### Notes

References: Schouten (1992). Biological data: Bivoltine.

#### Euchromius gozmanyi

Bleszynski, 1961

D5130CCD-8443-5558-A0BE-6264B050D341

##### Distribution

Atlanto-Mediterranean

##### Notes

Biological data: Bivoltine. Flight period: IV-VI, VIII-IX. First record in Murcia Region.

#### Euchromius ramburiellus

(Duponchel, 1836)

465F40C2-5F0F-5979-8759-41948D5C9B47

##### Distribution

Mediterranean-Asiatic

##### Notes

References: Caradja (1910), De Prins (1985), Schouten (1992). Biological data: Polyvoltine. Flight period: IV-X.

#### Euchromius gratiosella

(Caradja, 1910)

C31EB8ED-E6E7-5082-9F20-120AC9D4BBB5

##### Distribution

Eurasiatic

##### Notes

References: Schouten (1992). Biological data: Bivoltine. Flight period: IV-X.

#### Euchromius cambridgei

(Zeller, 1867)

9E9A552C-105A-5E2C-BB93-C52DEA9D9410

##### Distribution

Mediterranean-Asiatic

##### Notes

References: Fazekas (2017). Biological data: Bivoltine. Flight period: VI-X.

#### Chrysoteuchia culmella

(Linnaeus, 1758)

0111C0FB-AD2E-5989-8785-9E1C77AEE977

##### Distribution

Palaearctic

##### Notes

References: Zerny (1914). Biological data: Univoltine.

#### Angustalius malacellus

(Duponchel, 1836)

9A003E89-7A3B-55A0-A7E8-1E90AF2F5B94

##### Distribution

Mediterranean-Asiatic

##### Notes

Biological data: Univoltine. Flight period: V. First record in Murcia Region.

#### Agriphila tristella

([Denis & Schiffermüller], 1775)

8956F9E6-BAEE-5F03-AA80-CBE8C8F8DE89

##### Distribution

Eurasiatic

##### Notes

Biological data: Bivoltine. Flight period: IX. First record in Murcia Region.

#### Agriphila inquinatella

(Denis & Schiffermuller, 1775)

28272801-C21D-560E-9754-B8DFE6958529

##### Distribution

Eurasiatic

##### Notes

Biological data: Univoltine. Flight period: VIII. First record in Murcia Region.

#### Agriphila trabeatellus

(Herrich-Schäffer, 1848)

27554D61-65F6-58FB-AEA4-4EE2714C3B88

##### Distribution

Mediterranean-Asiatic

##### Notes

References: Bleszyński (1959). Biological data: Univoltine. Flight period: VIII-X.

#### Agriphila cyrenaicellus

(Ragonot, 1887)

7318109B-870E-5C5C-A30D-F09995004337

##### Distribution

Mediterranean-Asiatic

##### Notes

Biological data: Univoltine. Flight period: VIII-X. First record in Murcia Region.

#### Agriphila geniculea

(Haworth, [1841])

052BE30E-5A59-5402-83F2-7430932C7355

##### Distribution

Eurasiatic

##### Notes

References: Fazekas (2017). Biological data: Univoltine. Flight period: IX.

#### Catoptria pinella

(Linnaeus, 1758)

8142B5E6-73A6-5B2E-BED4-74BF6C080BAF

##### Distribution

Eurasiatic

##### Notes

Biological data: Univoltine. Flight period: VIII-IX. First record in Murcia Region.

#### Catoptria fulgidella

(Hübner, [1813])

E0013111-43AD-57E9-946E-E9A5367D1AD1

##### Distribution

Eurasiatic

##### Notes

Biological data: Univoltine. Flight period: IX. First record in Murcia Region.

#### Catoptria staudingeri

(Zeller, 1863)

B6653DBE-29F2-50CF-BA13-285A71D8FEDA

##### Distribution

Atlanto-Mediterranean

##### Notes

Biological data: Univoltine. Flight period: IX. First record in Murcia Region.

#### Mesocrambus pallidellus

(Duponchel, 1836)

54725F46-7D06-52A3-B35D-0C84481D7B9C

##### Distribution

Atlanto-Mediterranean

##### Notes

References: Bleszyński (1957), Derra and Hacker (1982). Biological data: Univoltine. Flight period: VII-IX.

#### Mesocrambus salahinellus

(Chrétien, 1917)

BB53B2EF-F5AA-5D33-A097-BB4C5209FF57

##### Distribution

Atlanto-Mediterranean

##### Notes

Biological data: Univoltine. Flight period: VI. First record in Murcia Region.

#### Xathocrambus delicatellus

(Zeller, 1863)

80C2368A-89A3-5A26-9186-529E7DB234D4

##### Distribution

Atlanto-Mediterranean

##### Notes

Biological data: Univoltine. Flight period: VI-IX. First record in Murcia Region.

#### Xanthocrambus caducellus

(Muller-Rutz, 1909)

4797CE31-3699-545E-81E9-330583128EC1

##### Distribution

Atlanto-Mediterranean

##### Notes

Biological data: Univoltine. Flight period: VI. First record in Murcia Region.

#### Chrysocrambus sardiniellus

(Turati, 1911)

F0B37680-A5F2-5BDB-B73C-0CA2D6BE6B6E

##### Distribution

Atlanto-Mediterranean

##### Notes

Biological data: Univoltine. Flight period: VI. First record in Murcia Region.

#### Pediasia contaminella

(Hübner, 1796)

8DA89A93-17E1-5187-9147-94C9E9BB442D

##### Distribution

Eurasiatic

##### Notes

Biological data: Bivoltine. Flight period: V-VII. First record in Murcia Region.

#### Pediasia ribbeella

(Caradja, 1910)

22BC28D0-14BD-5CA2-A43A-85BAFECC7FD6

##### Distribution

Endemic

##### Notes

References: Caradja (1916). Biological data: Univoltine. Flight period: V.

#### Pediasia serraticornis

(Hampson, 1900)

612478C8-4F32-59B4-A62E-12BA87088512

##### Distribution

Mediterranean-Asiatic

##### Notes

Biological data: Univoltine. Flight period: X. First record in Murcia Region.

#### Ancylolomia palpella

([Denis & Schiffermüller], 1775)

FDECBF45-10EB-5659-90F0-EC98CE6D9544

##### Distribution

Mediterranean-Asiatic

##### Notes

Biological data: Univoltine. Flight period: IX-X. First record in Murcia Region.

#### Ancylolomia tentaculella

(Hubner, 1796)

CA2D4285-2FCB-56E2-87D1-D081152D120C

##### Distribution

Mediterranean-Asiatic

##### Notes

References: Agenjo (1963). Biological data: Univoltine. Flight period: VIII-X.

#### Ancylolomia disparalis

(Hübner, 1825)

F0CA65C4-EC19-5915-BD4A-D0F136F33576

##### Distribution

Mediterranean-Asiatic

##### Notes

References: Caradja (1910), Agenjo (1963). Biological data: Univoltine. Flight period: IX-X.

#### Ancylolomia tripolitella

Rebel, 1909

5DAB1EC4-3084-5BCB-A8D3-E9E6BEC6E2F0

##### Distribution

Mediterranean-Asiatic

##### Notes

Biological data: Univoltine. Flight period: IX-X. First record in Murcia Region.

#### Pseudoctenella inornata

Staudinger, 1870

624C3D3A-F6D4-5A63-8EA4-50A59EF3FFEC

##### Distribution

Mediterranean-Asiatic

##### Notes

References: Agenjo (1963), Pérez de-Gregorio and Requena (2008). Biological data: Univoltine. Flight period: VIII-X.

#### Glaphyriinae

85F0479D-5440-5CFC-8037-C3B227BC3F85

#### Hellula undalis

(Fabricius, 1775)

23635E25-A208-5EE9-91F4-19FC327C3E56

##### Distribution

Cosmopolitan

##### Notes

Biological data: Bivoltine. Flight period: I-XII. First record in Murcia Region.

#### Evergestis frumentalis

(Linnaeus, [1760])

885E4C29-B31C-526D-A6E8-D66813E2EF64

##### Distribution

Eurasiatic

##### Notes

Biological data: Bivoltine. Flight period: III-VIII. First record in Murcia Region.

#### Evergestis desertalis

(Hübner, 1813)

2CBD0E2E-7C0D-52F1-91B2-4608F0A9BEF7

##### Distribution

Mediterranean-Asiatic

##### Notes

References: Caradja (1916), Agenjo (1952), De Prins (1985), Palacios and Abad (2010). Biological data: Bivoltine. Flight period: III-X.

#### Evergestis dusmeti

Agenjo, 1955

D36F7B6C-3EE6-5517-A703-2333CA924093

##### Distribution

Atlanto-Mediterranean

##### Notes

Biological data: Univoltine. Flight period: X-V. First record in Murcia Region.

#### Evergestis extimalis

(Scopoli, 1763)

132145BC-24D6-51BC-A74E-B88D53D10E88

##### Distribution

Eurasiatic

##### Notes

Biological data: Bivoltine. Flight period: X. First record in Murcia Region.

#### Evergestis marionalis

Leraut, 2003

A5A51C0B-DF73-52F8-8285-5AFA0F1F291D

##### Distribution

Atlanto-Mediterranean

##### Notes

Biological data: Bivoltine. Flight period: II, V, IX-X. First record in Murcia Region.

#### Evergestis politalis

([Denis & Schiffermüller], 1775)

3B3B6F30-6B6A-517F-9A8A-FEB4B9687047

##### Distribution

Eurasiatic

##### Notes

Biological data: Bivoltine. Flight period: IX-X. First record in Murcia Region.

#### Evergestis dumerlei

Leraut, 2003

7A74391A-7B96-5564-BC0E-FCBC32B83393

##### Distribution

Atlanto-Mediterranean

##### Notes

Biological data: Univoltine. Flight period: VII-XI. First record in Murcia Region.

#### Evergestis mundalis

(Guenée, 1854)

F3520B78-03F3-542C-8FAA-0F8D8AAF92B8

##### Distribution

Mediterranean-Asiatic

##### Notes

Biological data: Bivoltine. Flight period: VIII. First record in Murcia Region.

#### Evergestis isatidalis

(Duponchel, 1833)

391D2777-F209-53DD-95D4-C7C85858A097

##### Distribution

Mediterranean-Asiatic

##### Notes

References: Palacios and Abad (2010). Biological data: Univoltine. Flight period: VIII-IV.

#### Hyperlais lutosalis

(Mann, 1862)

27F29059-EA1B-598D-8F92-6B42CFEDA985

##### Distribution

Mediterranean-Asiatic

##### Notes

References: Agenjo (1952), Leraut (2012), Gastón et al. (2015). Biological data: Bivoltine. Flight period: V-VII, IX.

#### Lathrotelinae

CDD9A53E-0918-5F30-AFEA-596743058EEC

#### Diplopseustis perieresalis

(Walker, 1859)

5964DF54-C8B3-5640-B377-58AD1D36F0D1

##### Distribution

Tropical

##### Notes

Biological data: Polyvoltine. Flight period: X. First record in Murcia Region.

#### Odontiinae

EF109992-94C7-5FB9-B362-7D11570DE56C

#### Ephelis pudicalis

(Duponchel, [1832])

4CCBC27F-0591-5B3E-A2E6-9ED18B5678EE

##### Distribution

Atlanto-Mediterranean

##### Notes

References: Zerny (1914), Caradja (1916). Biological data: Univoltine. Flight period: V-VI.

#### Titanio tarraconensis

Leraut & Luquet, 1983

13C9EA29-EEB2-577D-9512-20965D9AEF80

##### Distribution

Atlanto-Mediterranean

##### Notes

References: Agenjo (1952), Slamka (2006). Biological data: Bivoltine. Flight period: IV-V.

#### Cynaeda dentalis

([Denis & Schiffermüller], 1775)

D2893E41-C14B-5178-B8A5-4EE171DC93E0

##### Distribution

Eurasiatic

##### Notes

Biological data: Bivoltine. Flight period: VI. First record in Murcia Region.

#### Tegostoma comparalis

(Hübner, 1796)

FC45F0E4-7EBC-531E-A65E-FDC3B5B99547

##### Distribution

Mediterranean-Asiatic

##### Notes

Biological data: Bivoltine. Flight period: VII. First record in Murcia Region.

#### Tegostoma erubescens

(Christoph, 1877)

4D55C0A6-618E-5B42-B434-6D67AC4CBFD7

##### Distribution

Mediterranean-Asiatic

##### Notes

References: De Prins (1985). Biological data: Univoltine. Flight period: VII-IX.

#### Aporodes floralis

(Hübner, 1809)

04FF62B6-F013-5614-8490-460938290E83

##### Distribution

Mediterranean-Asiatic

##### Notes

References: Agenjo (1952). Biological data: Bivoltine. Flight period: V-IX.

#### Pyraustinae

2CE7AFBF-2B5C-5097-886E-EE2BB3C83B50

#### Loxostege (Loxostege) scutalis

(Hübner, [1813])

10549CBB-E5E6-506D-BCBA-0EC80AB1ED9D

##### Distribution

Atlanto-Mediterranean

##### Notes

References: Slamka (2013). Biological data: Univoltine. Flight period: II-IV.

#### Loxostege (Loxostege) comptalis

(Freyer, [1848])

1E581297-055A-51BB-84DC-65B358210103

##### Distribution

Atlanto-Mediterranean

##### Notes

References: Zerny (1914), Caradja (1916), Derra and Hacker (1982), Slamka (2013). Biological data: Bivoltine. Flight period: II-VII, IX-X.

#### Loxostege (Loxostege) clathralis

(Hübner, [1813])

C2012B1A-8BE0-5355-9110-E5B49F3A6964

##### Distribution

Mediterranean-Asiatic

##### Notes

References: Zerny (1914). Biological data: Univoltine.

#### Loxostege (Margaritia) sticticalis

(Linnaeus, [1760])

3ED900BF-0DB2-5FCD-BEEF-08FB0DB2EDA5

##### Distribution

Holarctic

##### Notes

References: Agenjo (1952), Derra and Hacker (1982). Biological data: Bivoltine. Flight period: III-VII, IX-X.

#### Achyra nudalis

(Hübner, 1796)

A4423DC6-40DD-593F-A2FC-C678AA1DC230

##### Distribution

Tropical

##### Notes

References: Derra and Hacker (1982). Biological data: Bivoltine. Flight period: IV-IX.

#### Palepicorsia ustrinalis

(Christoph, 1877)

5B21D864-E6DD-5988-8D31-808BE2335B31

##### Distribution

Mediterranean-Asiatic

##### Notes

References: Palacios and Abad (2010). Biological data: Univoltine. Flight period: IV-VIII.

#### Paracorsia repandalis

([Denis & Schiffermüller], 1775)

5126C1C6-2457-546A-854A-6D02A6096483

##### Distribution

Holarctic

##### Notes

Biological data: Bivoltine. Flight period: V-VI, IX-X. First record in Murcia Region.

#### Ecpyrrhorrhoe diffusalis

(Guenée, 1854)

47F6B811-B9CC-5820-89E0-9330B8B36199

##### Distribution

Tropical

##### Notes

References: Agenjo (1952), Pérez de-Gregorio et al. (2011), Slamka (2013). Biological data: Polyvoltine. Flight period: II-X.

#### Pyrausta (Pyrausta) pellicalis

(Staudinger, 1870)

A340C378-C581-5912-8227-EA059AAD1575

##### Distribution

Atlanto-Mediterranean

##### Notes

Biological data: Univoltine. Flight period: V-VII. First record in Murcia Region.

#### Pyrausta (Pyrausta) sanguinalis

(Linnaeus, 1767)

91A2AF7C-E5B4-53F4-BAAD-155D78D126D9

##### Distribution

Eurasiatic

##### Notes

References: Agenjo (1952), Palacios and Abad (2010). Biological data: Bivoltine. Flight period: II-X.

#### Pyrausta (Pyrausta) despicata

(Scopoli, 1763)

5F826419-0778-5997-A914-C6A8D8B7290A

##### Distribution

Holarctic

##### Notes

Biological data: Bivoltine. Flight period: V-X. First record in Murcia Region.

#### Pyrausta (Pyrausta) acontialis

(Staudinger, 1859)

5942D919-4191-5744-9F61-63AE6EB86A40

##### Distribution

Mediterranean-Asiatic

##### Notes

Biological data: Univoltine. Flight period: III-IV. First record in Murcia Region.

#### Pyrausta (Pyrausta) aurata

(Scopoli, 1763)

F1CD8C78-5EB3-5094-8C63-6829B3EA2E25

##### Distribution

Palaearctic

##### Notes

Biological data: Bivoltine. Flight period: VII-VIII, X. First record in Murcia Region.

#### Pyrausta (Pyrausta) purpuralis

(Linnaeus, 1758)

E26D1F25-90FB-5D2D-8A78-C0F2A0DEE64E

##### Distribution

Eurasiatic

##### Notes

References: Agenjo (1974). Biological data: Bivoltine. Flight period: V.

#### Pyrausta (Pyrausta) ostrinalis

(Hubner, 1796)

DE54E143-C8A0-5506-A3C0-2FC1BDE016BE

##### Distribution

Eurasiatic

##### Notes

Biological data: Bivoltine. Flight period: IV. First record in Murcia Region.

#### Pyrausta (Panstegia) limbopunctalis

(Herrich-Schäffer, 1849)

D30AA2F5-4C8A-5474-9EEB-472D58767937

##### Distribution

Mediterranean-Asiatic

##### Notes

References: Slamka (2013). Biological data: Univoltine. Flight period: VI-IX.

#### Uresiphita gilvata

(Fabricius, 1794)

4562B51C-6631-55AA-B4BB-60A3DD5788A9

##### Distribution

Cosmopolitan

##### Notes

References: Derra and Hacker (1982). Biological data: Bivoltine. Flight period: IV-X.

#### Sitochroa palealis

([Denis & Schiffermüller], 1775)

B47F69D4-681D-5F1A-9E0E-C8DBC68BE315

##### Distribution

Holarctic

##### Notes

Biological data: Bivoltine. Flight period: II-IV, VI. First record in Murcia Region.

#### Euclasta varii

Popescu-Gorj & Constantinescu, 1973

EFE53C4D-9364-5ACC-8E42-B5A3BC408BF3

##### Distribution

Tropical

##### Notes

References: Palacios and Abad (2010). Biological data: Bivoltine. Flight period: IV-VI, VIII-X.

#### Ostrinia nubilalis

(Hübner, 1796)

13B052F2-B0AB-5395-AC54-A8C733F3E474

##### Distribution

Holarctic

##### Notes

References: Agenjo (1952), Slamka (2013). Biological data: Polyvoltine. Flight period: V, IX-X.

#### Anania (Anania) verbascalis

([Denis & Schiffermüller], 1775)

370EA742-C787-529F-B880-00DCD7827D2A

##### Distribution

Eurasiatic

##### Notes

Biological data: Bivoltine. Flight period: IX. First record in Murcia Region.

#### Anania (Ametasia) murcialis

(Ragonot, 1895)

38F10BEB-56CC-59A7-8250-D08449C39B8E

##### Distribution

Atlanto-Mediterranean

##### Notes

References: Ragonot (1895), Agenjo (1962). Biological data: Bivoltine. Flight period: V-VI, IX.

#### Anania (Ebulea) testacealis

(Zeller, 1847)

716FF492-053A-54E1-852C-622360C10773

##### Distribution

Mediterranean-Asiatic

##### Notes

References: Derra and Hacker (1982). Biological data: Bivoltine. Flight period: VII.

#### Scopariinae

05CFD085-5F13-57B4-B786-C14FA7D0AECC

#### Scoparia pyralella

([Denis & Schiffermüller], 1775)

07746DD2-01CB-5C3D-93D8-F30A011CB586

##### Distribution

Eurasiatic

##### Notes

Biological data: Univoltine. Flight period: VII. First record in Murcia Region.

#### Scoparia staudingeralis

(Mabille, 1869)

023BAE81-1571-54D6-939B-930977F86606

##### Distribution

Mediterranean-Asiatic

##### Notes

Biological data: Univoltine. Flight period: IV-VI. First record in Murcia Region.

#### Scoparia gallica

Peyerimhoff, 1873

43867B7B-932D-5833-8FDE-636C97C70B4F

##### Distribution

Mediterranean-Asiatic

##### Notes

Biological data: Univoltine. Flight period: VII. First record in Murcia Region.

#### Eudonia mercurella

(Linnaeus, 1758)

7D337BD1-0514-56EE-8B8F-E9654E1E89A1

##### Distribution

Eurasiatic

##### Notes

Biological data: Univoltine. Flight period: VII-VIII. First record in Murcia Region.

#### Eudonia angustea

(Curtis, 1827)

D58527B9-6AC8-5FF1-83DA-04F2A8FABBF2

##### Distribution

Mediterranean-Asiatic

##### Notes

Biological data: Bivoltine. Flight period: X-IV. First record in Murcia Region.

#### Eudonia lineola

(Curtis, 1827)

7F44F3D5-B68F-50B0-A4D5-0B6C62376DE7

##### Distribution

Atlanto-Mediterranean

##### Notes

Biological data: Bivoltine. Flight period: II-V, VIII, X, XII. First record in Murcia Region.

#### Spilomelinae

431B5E6B-51C5-514E-A50A-9B3D76A4FD34

#### Udea ferrugalis

(Hübner, 1796)

A7F889A6-2046-55ED-B18B-B82649D39052

##### Distribution

Cosmopolitan

##### Notes

References: Agenjo (1952), Palacios and Abad (2010). Biological data: Bivoltine. Flight period: I-XII

#### Udea institalis

(Hübner, 1819)

6727F9E0-9F51-557B-B809-7AD171C23B7E

##### Distribution

Mediterranean-Asiatic

##### Notes

References: Zerny (1914), Derra and Hacker (1982). Biological data: Univoltine. Flight period: VI-VIII.

#### Udea bipunctalis

(Herrich-Schäffer, 1851)

7F39CECD-D2B7-535C-BFB0-EB52D504133D

##### Distribution

Mediterranean-Asiatic

##### Notes

References: Zerny (1914). Biological data: Univoltine. Flight period: VI.

#### Udea numeralis

(Hübner, 1796)

1867F86D-A0FC-57B7-A2BF-29A5F155D1FF

##### Distribution

Mediterranean-Asiatic

##### Notes

References: Derra and Hacker (1982). Biological data: Bivoltine. Flight period: II-XI.

#### Udea simplicella

(La Harpe, 1861)

D52568E5-978E-5682-BB8D-0133F0E02A85

##### Distribution

Atlanto-Mediterranean

##### Notes

References: Girdley et al. (2021). Biological data: Univoltine. Flight period: IX-X.

#### Mecyna lutealis

(Duponchel, [1833])

44C4C148-C75C-512C-8722-C102D99F4304

##### Distribution

Mediterranean-Asiatic

##### Notes

Biological data: Univoltine. Flight period: V-VII. First record in Murcia Region.

#### Mecyna trinalis

([Denis & Schiffermüller], 1775)

1A0E5A0F-43DE-5526-8A32-1328327D033C

##### Distribution

Mediterranean-Asiatic

##### Notes

References: Zerny (1914), Caradja (1916), Pérez de-Gregorio (2006), Pérez de-Gregorio et al. (2011). Biological data: Univoltine. Flight period: VI-VII.

#### Mecyna auralis

(Peyerimhoff, 1872)

BD5605C9-269E-5410-B8D8-51F985F78226

##### Distribution

Atlanto-Mediterranean

##### Notes

References: Slamka (2010), Slamka (2013). Biological data: Univoltine. Flight period: VI-VIII.

#### Mecyna asinalis

(Hübner, 1819)

071F34FD-0440-5717-BA20-879840034D35

##### Distribution

Mediterranean-Asiatic

##### Notes

Biological data: Bivoltine. Flight period: I-IV, VIII, X, XII. First record in Murcia Region.

#### Diasemiopsis ramburialis

(Duponchel, [1834])

3F957A59-8047-570A-BAB5-97758B733CC0

##### Distribution

Tropical

##### Notes

References: Derra and Hacker (1982). Biological data: Bivoltine. Flight period: VII.

#### Duponchelia fovealis

Zeller, 1847

EB9195E6-5477-55B1-ADB5-10DD233E6601

##### Distribution

Cosmopolitan

##### Notes

References: Slamka (2010). Biological data: Bivoltine. Flight period: IV-X.

#### Dolicharthria punctalis

([Denis & Schiffermüller], 1775)

F91275B5-C15A-59B8-8397-CC57AA62BAFB

##### Distribution

Eurasiatic

##### Notes

Biological data: Bivoltine. Flight period: IV-VII, IX-X. First record in Murcia Region.

#### Dolicharthria bruguieralis

(Duponchel, 1833)

1F5493C6-48E2-5F34-B007-8B1100B6AF9D

##### Distribution

Tropical

##### Notes

References: Agenjo (1952), Palacios and Abad (2010). Biological data: Bivoltine. Flight period: IV-XI.

#### Antigastra catalaunalis

(Duponchel, 1833)

40C181C8-AE05-5467-821B-9E56666572F6

##### Distribution

Tropical

##### Notes

References: Palacios and Abad (2010). Biological data: Univoltine. Flight period: VII-XI.

#### Spoladea recurvalis

(Fabricius, 1775)

8B59E662-E1B9-5567-98CD-9E12DC3D2790

##### Distribution

Cosmopolitan

##### Notes

References: Palacios and Abad (2010). Biological data: Bivoltine. Flight period: VIII-XII.

#### Hodebertia testalis

(Fabricius, 1794)

4C5E1872-9324-58BB-A026-4AA61A29945E

##### Distribution

Tropical

##### Notes

References: Slamka (2010), Slamka (2013). Biological data: Univoltine. Flight period: VII-XI.

#### Palpita vitrealis

(Rossi, 1794)

F12B2150-0180-5831-9B7D-9653B46420B5

##### Distribution

Cosmopolitan

##### Notes

References: Derra and Hacker (1982). Biological data: Polyvoltine. Flight period: I-XII.

#### Hydriris ornatalis

(Duponchel, [1832])

41AD8E45-B814-53B8-AE56-22E4B65F80E5

##### Distribution

Cosmopolitan

##### Notes

Biological data: Bivoltine. Flight period: IV-XI. First record in Murcia Region.

#### Arnia nervosalis

Guenée, 1849

D29A1B2B-3BB7-5A6A-A280-1790B02E59B6

##### Distribution

Mediterranean-Asiatic

##### Notes

Biological data: Bivoltine. Flight period: IV- VI, IX-X. First record in Murcia Region.

#### Metasia (Metasia) suppandalis

(Hübner, 1823)

4E265F36-EF58-5BA2-B037-C83334538D0B

##### Distribution

Mediterranean-Asiatic

##### Notes

References: Derra and Hacker (1982), Pérez de-Gregorio and Requena (2010). Biological data: Univoltine. Flight period: V-IX.

#### Metasia (Metasia) hymenalis

Guenée, 1854

9EEF8CE7-AF90-5032-A361-F4A72ADA428D

##### Distribution

Atlanto-Mediterranean

##### Notes

References: Zerny (1914), Caradja (1916), Agenjo (1952), Derra and Hacker (1982), Slamka (2013). Biological data: Univoltine. Flight period: V-VIII.

#### Metasia (Metasia) corsicalis

(Duponchel, [1833])

DAE28722-3F4B-5696-8E8E-F97AF3F96227

##### Distribution

Mediterranean-Asiatic

##### Notes

References: Slamka (2013). Biological data: Univoltine. Flight period: VII-VIII.

#### Metasia (Metasia) ibericalis

(Ragonot, 1894)

56186551-21E0-5C99-824A-016A70FB1090

##### Distribution

Atlanto-Mediterranean

##### Notes

References: Slamka (2013). Biological data: Univoltine. Flight period: VI-VIII.

#### Metasia (Clasperia) cuencalis

Ragonot, 1894

58E239DB-2C3D-5910-B181-9D82B5D9D770

##### Distribution

Atlanto-Mediterranean

##### Notes

References: Zerny (1914), Palacios and Abad (2010), Slamka (2013). Biological data: Univoltine. Flight period: VI-IX.

#### Nomophila noctuella

([Denis & Schiffermüller], 1775)

8F3EC106-BF6A-52F5-81EC-4861DFE148DE

##### Distribution

Cosmopolitan

##### Notes

References: Palacios and Abad (2010). Biological data: Polyvoltine. Flight period: I-XII.

#### Herpetogramma licarsisalis

(Walker, 1859)

7A3E1944-BC6B-5FC1-B628-45F387851D58

##### Distribution

Tropical

##### Notes

Biological data: Univoltine. Flight period: X. First record in Murcia Region.

## Analysis

The list includes 106 species in 50 genera and 8 subfamilies: Acentropinae (one species), Crambinae (32 species), Glaphyriinae (11 species), Lathrotelinae (one species), Odontiinae (six species), Pyraustinae (23 species), Scopariinae (six species) and Spilomelinae (26 species). Forty nine new records from the Murcia Region are added to its Lepidopteran fauna.

The most species-rich subfamily Crambinae comprises 26% of all genera and 30.2% of all species, while Spilomelinae comprise 30% and 25.5%, followed by Pyraustinae (22% and 21.7%) and Glaphyriinae (2% and 10.3%), respectively (Table 1). The remaining subfamilies collectively constitute 18% and 13.2% of all genera and species known from the Murcia Region, respectively (10% and 5.7% for Odontiinae, 4% and 5.7% for Scopariinae and 2% and 0.9% for Acentropinae and Lathrotelinae, respectively; Table 1).

The European family of Crambidae consists of ca. 90 species (Leraut 2012), whilst the Iberian Crambidae fauna comprises 256 extant species (Vives-Moreno 2014). Thus, to date, the number of species known from the Murcia Region accounts for approximately 21.63% of the European total and 41.41% of the Iberian species.

Known Crambidae diversity in the Murcia Region seem relatively rich when compared to those in other Iberian Regions and with the whole of the Iberian Peninsula, as for instance, nearby areas like the Natural Park of Cabo de Gata-Nijar in Almeria (51 species; Garre et al. 2018) or more extensive Iberian Regions, such as Catalonia (186 species; in Dantart 2019) and Aragon (181 species; Redondo et al. 2017). This may be because intensive surveys have started only recently or because the biodiversity is greater closer to the temperate areas.

Knowledge on Crambidae diversity in Murcia Region is still incomplete, but is probably even more limited in nearby Regions, with less than 65 species recorded in littoral wetlands in Catalonia (Pérez de-Gregorio 2001), coastal wetlands and saltmarshes in Huelva (Huertas 2007) and the mountainous area of Ports de Tortosa-Beseit (Pérez de-Gregorio 2008).

The most species-rich Crambidae genera in the Murcia Region are *Evergestis* (9 species, 8.49%), *Pyrausta* (8 species, 7.55%), *Euchromius* (6 species, 5.66%), *Metasia*, *Udea* and *Agriphila* (5 species, 4.72%) and *Ancylolomia*, *Mecyna* and *Loxostege* (4 species, 3.77% each, respectively). The majority of genera (10) are species-poor (2-3 species) or known in the Murcia Region from a single species (31 genera).

Species richness varies substantially amongst the different bioclimatic areas of the Murcia Region (Fig. 1). The Thermomediterranean area has the most diverse Crambidae fauna with 69 species recorded, followed by the Mesomediterranean area with 59 species, while the Supra- and Oromediteranean areas appear to be less diverse with 29 species (Table 2). In each of these areas, 29 species are unique in the Thermo-, 12 in Meso- and 9 in Supra- and Oromediterranean areas, while 38 species were recorded in two areas and 10 in the three studied areas.

Approximately half of the species can be considered specialists in a given bioclimatic area, while the other 50% can be considered as opportunists of different types of vegetation that characterise each of the bioclimatic areas. The detailed data for the bioclimatic areas of Crambidae in the Murcia Region are summarised in Table 2.

Chorological analysis for the family Crambidae in the Region of Murcia showed that the Mediterranean chorotype, including the endemic *Pediasiaribbeellus* (Caradja), is the most abundant with 56.6% of the total, which is consistent with the geographical position of the study area. Amongst these, the Asiatic-Mediterranean elements (34.9%) are more frequent than the Atlanto-Mediterranean elements (21.7%). On the other hand, the elements of wide distribution, such as the Eurasiatic, Holarctic and Palaearctic (26.4%), are the most common in the mountainous biotopes of the centre and north of the study area, while the tropical and cosmopolitan species (17.0%) have their origin mainly in Africa. The presence of opportunistic species is due to the agricultural crop fields that dominate a part of the Murcian territory.

Regarding the biology of the species, the environmental conditions of the study area, which affect the availability of trophic resources for reproduction, suggest that most of the species are bivoltins (47.2%) and univoltins (47.2%), while the rest are polyvoltins (5.7%). Most of the recorded species feed on plant species belonging to the Brassicaceae, Asteraceae, Lamiaceae, Chenopodiaceae, Scrophulariaceae and Amaranthaceae families, amongst others, although the species of the Crambinae subfamily feed on grasses (Poaceae). The most particular cases are those related to the genus *Eudonia* which feed on lichens and the species *Euchromiusocellea*, *E.cambridgei* and *Dolicharthriabruguieralis* which feed on plant detritus. Some species, such as *Palpitavitrealis*, *Ostrinianubilalis* and *Nomophilanoctuella*, must be controlled since they are agricultural crop pests. Finally, the host plants of 34.0% of species are unknown, so it will be necessary to carry out complementary studies to further biological understanding.

## Discussion

Prior to our investigation, the number of known Crambidae moth species in the Murcia Region was 56. Our study increases this number to a total of 106, based on an examination of museum specimens, published records and sampled individuals, 41.41% of all of the Iberian species known. This study presents an updated checklist of current Crambidae moth species with their distribution and biological information for the Murcia Region in the south-eastern Iberian Peninsula.

This study serves as both a guide for collection in the poorly sampled south-western European continent and a comprehensive reference list with the Crambidae taxa and localities where conservation is an important priority for policy-makers, conservation planners and for the management of insect diversity in Spain.

We encourage lepidopterists holding additional data on systematically collected crambids to produce an updated dataset. Additionally, new intensive surveys in adjacent regions are being conducted, as well as unknown specimens being continuously identified to species level.

## Figures and Tables

**Table 1.** Numbers and percentages of known genera and species recorded for each subfamily in Murcia Region.

**Subfamilies**	**Genus richness**	% **Genus**	**Species richness**	% **Species**
Crambinae	13	26	32	30.2
Spilomelinae	14	30	26	25.5
Pyraustinae	11	22	23	21.7
Glaphyriinae	3	2	11	10.3
Odontiinae	5	10	6	5.7
Scopariinae	2	4	6	5.7
Acentropinae	1	2	1	0.9
Lathrotelinae	1	2	1	0.9
**Total**	**50**	**100**	**106**	**100**

**Table 2.** List of unique species in each bioclimatic area or in more than one bioclimatic area.

Oro- and Supramediterranean	*Agriphilainquinatella* (Denis & Schiffermuller, 1775)
	*Chrysocrambussardiniellus* (Turati, 1911)
	*Xanthocrambuscaducellus* (Muller-Rutz, 1909)
	*Evergestismundalis* (Guenée, 1854)
	*Pyraustaostrinalis* (Hubner, 1796)
	*Scopariagallica* Peyerimhoff, 1873
	*Scopariapyralella* ([Denis & Schiffermüller], 1775)
	*Mecynalutealis* (Duponchel, [1833])
	*Udeabipunctalis* (Herrich-Schäffer, 1851)
Mesomediterranean	*Agriphilageniculea* (Haworth, [1841])
	*Agriphilatristella* ([Denis & Schiffermüller], 1775)
	*Ancylolomiapalpella* ([Denis & Schiffermüller], 1775)
	*Angustaliusmalacellus* (Duponchel, 1836)
	*Catoptriafulgidella* (Hübner, [1813])
	*Catoptriapinella* (Linnaeus, 1758)
	*Catoptriastaudingeri* (Zeller, 1863)
	*Tegostomacomparalis* (Hübner, 1796)
	*Pyraustaacontialis* (Staudinger, 1859)
	*Diasemiopsisramburialis* (Duponchel, [1834])
	*Metasiahymenalis* Guenée, 1854
	*Udeainstitalis* (Hübner, 1819)
Thermomediterranean	*Ancylolomiadisparalis* (Hübner, 1825)
	*Chiloluteella* (Motschulsky, 1866)
	*Euchromiuscambridgei* (Zeller, 1867)
	*Euchromiusgozmanyi* Bleszynski, 1961
	*Euchromiusgratiosella* (Caradja, 1910)
	*Euchromiusocellea* (Haworth, 1811)
	*Mesocrambussalahinellus* (Chrétien, 1917)
	*Pediasiaribbeella* (Caradja, 1910)
	*Pediasiaserraticornis* (Hampson, 1900)
	*Pseudobissetiaterrestrellus* (Christoph, 1885)
	*Pseudoctenellainornata* Staudinger, 1870
	*Hyperlaislutosalis* (Mann, 1862)
	*Evergestisextimalis* (Scopoli, 1763)
	*Evergestispolitalis* ([Denis & Schiffermüller], 1775)
	*Cynaedadentalis* ([Denis & Schiffermüller], 1775)
	*Tegostomaerubescens* (Christoph, 1877)
	*Ananiamurcialis* (Ragonot, 1895)
	*Ananiaverbascalis* ([Denis & Schiffermüller], 1775)
	*Euclastavarii* Popescu-Gorj & Constantinescu, 1973
	*Loxostegescutalis* (Hübner, [1813])
	Pyrausta (Pyrausta) aurata (Scopoli, 1763)
	*Sitochroapalealis* ([Denis & Schiffermüller], 1775)
	*Arnianervosalis* Guenée, 1850
	*Diplopseustisperieresalis* (Walker, 1859)
	*Duponcheliafovealis* Zeller, 1850
	*Herpetogrammalicarsisalis* (Walker, 1859)
	*Metasiacorsicalis* (Duponchel, [1833])
	*Spoladearecurvalis* (Fabricius, 1775)
	*Udeasimplicella* (La Harpe, 1861)
Oro-, Supra- and Mesomediterranean	*Ephelispudicalis* (Duponchel, [1832])
	*Paracorsiarepandalis* ([Denis & Schiffermüller], 1775)
	*Pyraustalimbopunctalis* (Herrich-Schäffer, 1849)
	*Pyraustapellicalis* (Staudinger, 1870)
	*Scopariastaudingeralis* (Mabille, 1869)
	*Mecynaauralis* (Peyerimhoff, 1872)
	*Mecynatrinalis* ([Denis & Schiffermüller], 1775)
	*Metasiacuencalis* Ragonot, 1894
Meso- and Thermomediterranean	*Agriphilacyrenaicellus* (Ragonot, 1887)
	*Ancylolomiatentaculella* (Hubner, 1796)
	*Ancylolomiatripolitella* Rebel, 1909
	*Euchromiusramburiellus* (Duponchel, 1836)
	*Mesocrambuspallidellus* (Duponchel, 1836)
	*Pediasiacontaminella* (Hübner, 1796)
	*Evergestisdesertalis* (Hübner, 1813)
	*Evergestisdusmeti* Agenjo, 1960
	*Evergestisfrumentalis* (Linnaeus, [1760])
	*Evergestisisatidalis* (Duponchel, 1833)
	*Evergestismarionalis* Leraut, 2003
	*Aporodesfloralis* (Hübner, 1809)
	*Achyranudalis* (Hübner, 1796)
	*Loxostegecomptalis* (Freyer, [1848])
	*Loxostegesticticalis* (Linnaeus, [1760])
	*Ostrinianubilalis* (Hübner, 1796)
	*Palepicorsiaustrinalis* (Christoph, 1877)
	*Eudoniaangustea* (Curtis, 1827)
	*Eudonialineola* (Curtis, 1827)
	*Dolicharthriabruguieralis* (Duponchel, 1833)
	*Dolicharthriapunctalis* ([Denis & Schiffermüller], 1775)
	*Hodebertiatestalis* (Fabricius, 1794)
	*Hydririsornatalis* (Duponchel, [1832])
	*Mecynaasinalis* (Hübner, 1819)
	*Metasiaibericalis* (Ragonot, 1894)
	*Metasiasuppandalis* (Hübner, 1823)
	*Nomophilanoctuella* ([Denis & Schiffermüller], 1775)
	*Palpitavitrealis* (Rossi, 1794)
Oro- and Supra- and Thermomediterranean	*Agriphilatrabeatellus* (Herrich-Schäffer, 1848)
	*Antigastracatalaunalis* (Duponchel, 1833)
All areas	*Xathocrambusdelicatellus* (Zeller, 1863)
	*Evergestisdumerlei* Leraut, 2003
	*Hellulaundalis* (Fabricius, 1775)
	*Ecpyrrhorrhoediffusalis* (Guenée, 1854)
	*Pyraustadespicata* (Scopoli, 1763)
	*Pyraustasanguinalis* (Linnaeus, 1767)
	*Uresiphitagilvata* (Fabricius, 1794)
	*Eudoniamercurella* (Linnaeus, 1758)
	*Udeaferrugalis* (Hübner, 1796)
	*Udeanumeralis* (Hübner, 1796)
